# Supplementary material for: Environmental Substances Associated with Chronic Obstructive Pulmonary Disease—A Scoping Review
Source: Int J Environ Res Public Health. 2022 Mar 25;19(7):3945. doi: 10.3390/ijerph19073945 (PMC8997594; doi:10.3390/ijerph19073945)
Supplement: Supplementary file 1 [file ijerph-19-03945-s001.zip › ijerph-1600190-supplementary.pdf]

International Journal of
Environmental Research and Public Health

MDPI

Table S1. Pesticides studies on COPD.

Author, Year, and Title	Outcome Measured	Population Studied	Included Pesticides	Results
Pourhassan et al., 2019: Risk of obstructive pulmonary diseases and occupational exposure to pesticides: a systematic review and meta-analysis [21]	Review article, outcomes varied	Study designs and populations varied; 9 studies in occupational settings (8 cohort- and 1 case-control study; total N= 101 353)	E.g. insecticides, herbicides, and fungicides (as whole group)	A direct relationship between occupational exposure to pesticides and obstructive pulmonary diseases (odds ratio (OR)= 1.33, 95% confidence interval (CI) 1.21-1.47) was observed. There were positive significant relationships between occupational exposure to pesticides and risk of chronic bronchitis (OR= 1.27, 1.23-1.31) and an increased risk of COPD (OR= 1.44, 1.14-1.81). By the type of pesticides, a significant relationship was detected between occupational exposure to herbicides and increased risk of chronic bronchitis (OR= 1.28, 1.18-1.40) and risk of COPD (OR= 1.34, 1.12-1.59). Also, insecticides exposure was associated with chronic bronchitis (OR= 1.27, 1.20-1.35) and COPD (1.33, 1.05-1.70).
Doust et al., 2014: Is pesticide exposure a cause of obstructive airways disease? [22]	Review article, outcomes varied	Study designs and populations varied; 23 studies (17 cross-sectional, 2 case-control, and 3 cohort studies, and 1 systematic review). Six of the studies were agricultural occupational studies on airway obstruction (asthma and COPD), bronchitis, or wheezing.	E.g. organophosphate and carbamate insecticides, and paraquat	Exposure to pesticides may be associated with COPD. The strength of evidence for an association was weaker for COPD than for asthma. In three studies, significant associations were observed between pesticide exposure and bronchitis or COPD; of these studies, in Faria et al., 2005 [77] and Hoppin et al., 2007 [78], significant associations both with pesticides generally and with organophosphate exposure specifically. Significant associations detected with organophosphate and carbamate pesticides in the study by Chakraborty et al., 2009 [79]. A nonsignificant positive association with paraquat exposure was shown in the fourth study by Schenker et al., 2004 [80].
Fontana et al., 2017: Chronic Obstructive Pulmonary Disease in Farmers: A systematic review [9]	Review article, outcomes varied	Study designs and populations varied; 14 case-control or cohort studies. Three of the studies focused on	Organophosphate and carbamate insecticides, and paraquat	Between the studies, the prevalence of COPD varied from 3% to 68%. Livestock farmers were at higher risk. In one case-

		pesticides and COPD prevalence.		control study, the prevalence of COPD was significantly higher among farmers chronically exposed to organophosphates and carbamates compared to controls (18.1% vs. 6.9%) Furthermore, the severity of the disease was greater in farmers (9.0% vs. 4.0% for mild, 5.0% vs. 2.3% for moderate, and 2.9% vs. 0.5% for severe COPD forms). A positive association between red blood cell acetylcholinesterase inhibition and COPD prevalence was detected, which suggested an important causative role of pesticide exposures. A significantly increased risk of COPD (OR= 1.59, 95% CI 1.32-2.28) was observed among farmers in the study by Chakraborty et al., 2009 [79]. In a cohort study by De Jong et al., 2014 [81], higher risks of airway obstruction were detected among participants exposed to high levels of herbicides compared to controls (OR= 2.11, 1.03-4.30).
Ye et al., 2013: Occupational pesticide exposures and respiratory health [23]	Review article, outcomes varied	Study designs and populations varied; case-control or cross-sectional studies	Organophosphate and carbamate insecticides, organochlorines, and herbicides	The included studies strongly suggested an adverse effect of pesticide exposures on human respiratory health in occupational settings. Respiratory diseases including COPD were associated with occupational pesticide exposures. A higher prevalence of chronic bronchitis was associated with organophosphates and carbamate pesticide exposure (OR= 2.54, 95% CI 1.48-3.74) [79]. Organochlorine, organophosphate, carbamate pesticides, and herbicides were significantly associated with chronic bronchitis. Farmers with a history of high exposures to pesticides showed a higher prevalence of chronic bronchitis (OR= 1.85, 1.51-2.25) [78]. Incidence of chronic bronchitis among female non-smoking farmers was significantly related to

				application of five pesticides, such as insecticides dichlorvos (OR= 1.63, 1.01-2.61), herbicides cyanazine (OR= 1.88, 1.00-3.54), and paraquat (OR= 1.91, 1.02-3.55) [82].
Tarmure et al., 2020: Influence of pesticides on respiratory pathology - a literature review [24]	Review article, outcomes varied	Study designs and populations varied	E.g. organophosphates, fungicides, and herbicides	Occupational exposure to pesticides was correlated with numerous respiratory pathologies, including COPD. There was a positive association between exposure to specific insecticides, such as organophosphates, and chronic bronchitis in an agricultural health study on farmers; exposure to pesticides was associated with diagnosed chronic bronchitis (OR= 1.59, 95% CI 1.16-2.18) [83].
Ratanachina et al., 2020: Pesticide exposure and lung function: a systematic review and meta-analysis. [25]	Review article, outcomes varied	Study designs and populations varied	E.g. cholinesterase (ChE)-inhibiting pesticides, pyrethroids, dichlorodiphenyltrichloroethan (DDT), unspecified household insecticides, and herbicides (e.g. paraquats)	There was tentative evidence that exposure to ChE-inhibiting pesticides reduced forced expiratory volume in one second/forced vital capacity (FEV_1/FVC) in farmers.

The study of *Hoppin et al. (2007)* [78] was included in the studies of Doust et al. (2014) [22], Pourhassan et al. (2019) [21], Ye et al. (2013) [23], and Tarmure et al. (2020) [24]. The study of *Chakraborty et al. (2009)* [79] was included in the studies of Doust et al. (2014) [22], Fontana et al. (2017) [9] Ye et al. (2013) [23], and Ratanachina et al. (2020) [25]. The study of *Schenker et al. (2004)* [80] was included in the studies of Doust et al. (2014) [22] and Ratanachina et al. (2020) [25]. Finally, the study of *De Jong et al. (2014)* [81] was included in the studies of Fontana et al. (2017) [9] and Ratanachina et al. (2020) [25].

Table S2. Cd studies on COPD.

Author, Year, and Title	Outcome Measured	Population Studied	Results
Torén et al., 2019: The association between cadmium exposure and chronic airflow limitation and emphysema: the Swedish CArdioPulmonary BioImage Study (SCAPIS pilot) [17]	FEV_1/FVC, diffusing capacity (DLCO), total lung capacity (TLC), levels of Cd in blood (B-Cd), pulmonary computed tomography (CT)	A random population sample of 50-64-year-old study subjects (N= 1 111). Complete data on lung CT, lung function, and B-Cd from 741 individuals (381 men and 360 women, mean age 57 years).	Significant negative correlations were detected between B-Cd and FEV_1/FVC (r= -0.08), TLC (r= -0.25), and DLCO (r= -0.39). There were increased odds for emphysema (OR= 5.5, 95% CI 1.4-21.0), chronic airway limitation according to Global Initiative for Chronic Obstructive Lung Disease (GOLD) (OR= 2.8, 1.02-7.5), and chronic airway limitation defined as FEV_1/FVC (OR= 3.1, 1.1-8.4) in the highest B-Cd quartile compared to the lowest quartile.
Hassan et al., 2014: Accumulation of metals in GOLD4 COPD lungs is associated with decreased CFTR levels [31]	Expression of Cystic Fibrosis Transmembrane conductance Regulator (CFTR), cigarette smoke metal content in lung samples	A case-control study of COPD patients (GOLD 4) (N= 11) and healthy controls (GOLD 0) (N= 9)	Cd content in lung samples was significantly higher in GOLD 4 COPD patients in comparison to control smokers (GOLD 0) (p= 0.007).
Oh et al., 2014: Blood cadmium levels are associated with a	Prevalence of COPD, B-Cd levels	Cross-sectional data from the Korean National Health and Nutrition Examination Survey	There was an increasing prevalence of COPD in men (p for trend< 0.001), but not in

decline in lung function in males [34]		(KHANES) (2008-2011) (N= 3 622) among general population	women (p for trend= 0.67). A higher B-Cd level, within the normal range, was associated with COPD in men, including those who had never smoked (p for trend < 0.001 and p for trend= 0.008). There was no significant association between B-Cd levels and COPD in women (p for trend= 0.39 and p for trend= 0.43).
Skalny et al., 2020: Toxic metal exposure as a possible risk factor for COVID-19 and other respiratory infectious diseases [30]	Review article, outcomes varied	Study designs and populations varied	Heavy metal exposure, including Cd, was associated with respiratory dysfunction (reduced FVC, FEV_1) and respiratory diseases (COPD, bronchitis).
Gogoi et al., 2019: Circulatory heavy metals (cadmium, lead, mercury, and chromium) inversely correlate with plasma GST activity and GSH level in COPD patients and impair NOX4/Nrf2/GCLC/GST signaling pathway in cultured monocytes [32]	FEV_1/FVC, levels of heavy metals in plasma	A case-control study of COPD patients of coal mine site (N= 93) and healthy controls (N= 85)	Plasma Cd levels were significantly higher (p< 0.05) in COPD patients of coal mine site than in healthy controls. Plasma Cd levels in COPD patients were significantly and inversely correlated with lung function (FEV_1/FVC, predicted) (p< 0.001).
Leem et al., 2015: Relationship between blood levels of heavy metals and lung function based on the Korean National Health and Nutrition Examination Survey IV-V [33]	Lung function: FEV_1, FVC, FEV_1/FVC, levels of heavy metals in blood	Cross-sectional data from the KHANES 2008-2012 (N= 5 972) among general population. 674 (11.3%) subjects out of 5 972 were classified into the group having airway obstruction in spirometry.	FEV_1/FVC was significantly lower in the highest quartile than in the lowest quartile group of Cd concentration (78.3% vs. 79.2%, p< 0.001). An inverse relationship between the FEV_1/FVC and concentrations of Cd (estimated –0.005; p= 0.001) was detected. In current smokers, the risk of obstruction was higher in subjects in the highest quartile group of Cd concentration in comparison to those in the lowest quartile group (OR 1.94, 95% CI 1.06-3.57).

The study of *Oh et al. (2014)* [34] was also included in the study of Skalny et al. (2020) [30].

Table S3. Cr studies on COPD.

Author, Year, and Title	Outcome Measured	Population Studied	Results
Valdés et al., 2012: Elemental concentrations of ambient particles and cause specific mortality in Santiago, Chile: a time series study [39]	Particulate matter (PM2.5) and elements concentration, including Cr, on cause-specific mortality including COPD	Residents in the centre of Santiago, Chile	The strongest effect was found for the two-day average of PM2.5 and COPD mortality with 1.94% increase (95% CI 0.63-3.27) per 10μg/m³ increase in PM2.5.
Gogoi et al., 2019: Circulatory heavy metals (cadmium, lead, mercury, and chromium) inversely correlate with plasma GST activity and GSH level in COPD patients and impair NOX4/Nrf2/GCLC/GST signaling pathway in cultured monocytes [32]	FEV_1/FVC, levels of heavy metals in plasma	A case-control study of COPD patients of coal mine site (N= 93) and healthy controls (N= 85)	Plasma Cr levels were significantly higher (p< 0.05) in COPD patients of coal mine site than in healthy controls. Plasma Cr levels in COPD patients were significantly and inversely correlated with lung function (FEV_1/FVC, predicted) (p< 0.001).
Nasirzadeh et al., 2021: Health risk assessment of occupational exposure to Hexavalent Chromium in Iranian Workplaces: a meta-analysis study [36]	Meta-analysis, outcomes varied; occupational exposure to Cr(VI)	Study designs and populations varied; 11 case-control and 3 cross-sectional studies	All parameters of lung function were decreased in the Cr(VI)-exposed workers, especially FVC and FEV_1, in comparison to controls. Significant differences observed between lung function in case and control groups (p< 0.001). Exposure to Cr(VI) fume

			can cause COPD, which might occur in welders. Levels of Cr(VI) in urinary and blood samples were higher than threshold limit.

Table S4. As studies on COPD.

Authors, Year, and Title	Outcome Measured	Population Studied	Results
Smith et al., 2006: Increased mortality from lung cancer and bronchiectasis in young adults after exposure to arsenic in utero and in early childhood [42]	Long-term mortality impact of early life As exposure: the standardised mortality ratio (SMR) for bronchiectasis, levels of As in drinking water	Cohorts of young adults exposed to As in utero and early childhood, aged 30-49 at the time of death	Exposure to As in drinking water in early childhood or in utero caused pulmonary effects and increased subsequent mortality from COPD in young adults.
Parvez et al., 2013: Arsenic exposure and impaired lung function. Findings from a large population-based prospective cohort study [43]	Well water and urinary As concentrations, pulmonary function (FEV_1/FVC)	950 individuals having respiratory symptoms from a population-based cohort of 20 033 adults	For every standard deviation (SD) increase in baseline water As exposure, there was a lower level of FEV_1 (-46.5 ml, $p< 0.005$ and FVC (-53.1 ml, $p< 0.01$) detected. There were similar inverse relationships between baseline urinary As and FEV_1 (-48.3 ml, $p< 0.005$) and FVC (-55.2 ml, $p< 0.01$). A dose-related decrease in lung function with increasing levels of baseline water and urinary As was found; the association was significant even in never-smokers (N= 417) and subjects without skin lesions (N= 853), and stronger in men smokers (N= 447). In men smokers and individuals with skin lesions, every SD increase in water As was associated with a significant reduction in FEV_1 (-74.4 ml, $p< 0.01$ and -116.1 ml, $p< 0.05$) and FVC (-72.8 ml, $p= 0.02$ and -146.9 ml, $p= 0.004$), respectively.
Khan et al., 2020: Prospective cohort study of respiratory effects at ages 14 to 26 following early life exposure to arsenic in drinking water [15]	Lung function: FEV_1, FVC, FEV_1/FVC, respiratory symptoms, early life exposure to As in drinking water	A prospective cohort of 463 young adults (229 men and 234 women) aged 14-26 years; in the cohort, one group of children had been exposed to high As concentrations in drinking water in utero and early childhood (average 436 μg/L) and the other group of children were never known to have been exposed to >10 μg/L.	In the earlier study of the same cohort, significant increases in chronic respiratory symptoms in both boys and girls were detected. Similar findings were observed in this current follow-up study, though only in men. In men, FVC was reduced in the early-life high-exposure group in comparison to those never exposed (-95 ml, $p= 0.04$).
Sanchez et al., 2016: Inorganic arsenic and respiratory health, from early-life exposure to sex-specific effects: A systematic review [45]	Review article, outcomes varied. 29 studies included (16 cross-sectional, 8 longitudinal, 4 ecological, and 1 case-control study) investigating the relationship between inorganic As exposure and respiratory outcomes: i.e. lung function 9 studies, respiratory symptoms 18 studies, chronic non-malignant lung diseases 6 studies, and non-malignant lung disease mortality 5 studies	Both children and adults	Strong evidence was observed of a general association between As and non-malignant respiratory disease, including coherent evidence on lung function impairment and non-malignant lung disease mortality. Early life exposure (i.e. in utero and/or early childhood) had a significant effect through the lifespan.
Sanchez et al., 2018: A meta-analysis of arsenic exposure and lung function: Is there evidence of restrictive or obstructive lung disease? [44]	Meta-analysis of 9 studies. Lung function: FEV_1, FVC, FEV_1/FVC, levels of As in water	Total number of participants was 4 699, both children and adults	The pooled estimated mean difference (MD) comparing the highest category of As exposure versus the lowest for each study for FEV_1 was -42 mL (95% CI -70

			- -16) and for FVC -50 mL (-63 - - 37). Three of the studies presented effect estimates for FEV_1/FVC, for which there was no evidence of association. Arsenic was associated with restrictive impairments based on inverse associations between As and FEV_1 and FVC, though not with FEV_1/FVC, meaning that As exposure is associated with restrictive lung deficits.
Skalny et al., 2020: Toxic metal exposure as a possible risk factor for COVID-19 and other respiratory infectious diseases [30]	Review article, outcomes varied	Study designs and populations varied	Heavy metal exposure, including As, was associated with respiratory dysfunction (reduced FVC, FEV_1) and respiratory diseases (COPD, bronchitis).

The study of *Parvez et al. (2013)* [43] was also included in the study of Sanchez et al. (2018) [44]. The studies of *Khan et al. (2020)* [15] and *Sanchez et al. (2018)* [44] were also included in the study of Skalny et al. (2020) [30].

Table S5. Pb studies on COPD.

Authors, Year and Title	Outcome Measured	Population Studied	Results
Skalny et al., 2020: Toxic metal exposure as a possible risk factor for COVID-19 and other respiratory infectious diseases [30]	Review article, outcomes varied	Study designs and populations varied	Heavy metal exposure, including Pb, was associated with respiratory dysfunction (reduced FVC, FEV_1) and respiratory diseases (COPD, bronchitis).
Gogoi et al., 2019: Circulatory heavy metals (cadmium, lead, mercury, and chromium) inversely correlate with plasma GST activity and GSH level in COPD patients and impair NOX4/Nrf2/GCLC/GST signaling pathway in cultured monocytes [32]	FEV_1/FVC, levels of heavy metals in blood (plasma)	A case-control study of COPD patients of coal mine site (N= 93) and healthy controls (N= 85)	The blood levels of heavy metals, including Pb, were significantly higher ($p< 0.05$) in COPD patients of coal mine site in comparison to the healthy controls. The levels of the circulating heavy metals in COPD patients were significantly and inversely correlated with lung function (FEV_1/FVC, predicted) ($p< 0.001$).
Leem et al., 2015: Relationship between blood levels of heavy metals and lung function based on the Korean National Health and Nutrition Examination Survey IV-V [33]	Lung function: FEV_1, FVC, FEV_1/FVC, levels of heavy metals in blood	Cross-sectional data from the KHANES 2008-2012 (N= 5 972) among general population. 674 (11.3%) subjects out of 5 972 were classified into the group having airway obstruction in spirometry.	The FEV_1/FVC was lower in the highest quartile group of Pb (78.4% vs. 79.0%; $p= 0.025$) concentrations in comparison to those in the lowest quartile groups. There was an inverse relationship between the FEV_1/FVC and concentrations of Pb (estimated -0.002; $p= 0.007$).
Pan et al., 2020: Effects of Lead, Mercury, and Cadmium Co-exposure on Children's Pulmonary Function [47]	FEV_1, FVC, percent predicted FVC and FEV_1, levels of heavy metals in blood	A cross-sectional study of 221 healthy children (105 boys and 116 girls)	There was a significant positive interaction between the blood Pb levels on FVC ($\beta= 13.11$, $p= 0.03$), percent-predicted FVC ($\beta= 15.18$, $p= 0.04$), and percent-predicted FEV_1 ($\beta= 3.90$, $p= 0.03$). A borderline statistically significant positive interaction was detected between the blood Pb levels on FEV_1 ($\beta= 12.07$, $p= 0.09$). Only the blood Pb levels were significantly negatively associated with the FVC ($\beta= -4.83$, $p= 0.03$), FEV_1 ($\beta= -6.23$, $p= 0.02$), and percent predicted FEV_1 ($\beta= -5.39$, $p= 0.02$). The blood Pb levels were borderline significantly negatively associated with percent predicted FVC ($\beta= -5.49$, $p= 0.09$).

The study of *Gogoi et al. (2019)* [32] was also included in the study of Skalny et al. (2020) [30].

Table S6. Diisocyanates studies on COPD.

Authors, Year and Title	Outcome Measured	Population Studied	Results
Wegman et al., 1977: Chronic pulmonary function loss from exposure to toluene diisocyanate [52]	Acute pulmonary function changes: FEV_1	N= 112 workers in cushion manufactory exposed to TDI	The study population was divided into three different exposure subgroups (≤ 0.0015, 0.0020-0.0030, and ≥ 0.0035 ppm). FEV_1 of the highest exposure group fell 206 ml in two years (103 ml in a year), exceeding the expected value by three- to four-fold. There was a significant association ($r= 0.35$, $p< 0.005$) between acute and chronic decrement in FEV_1.
Alegre et al., 1990: Respiratory symptoms and pulmonary function of workers exposed to cork dust, toluene diisocyanate, and conidia [53]	Spirometry values: FEV_1, FVC	N= 442 cork processing workers, a cross-sectional study	A significant correlation was found between lengths of exposure to cork dust (polishers and grinders), TDI, conidia (slicers and punchers of mouldy cork), and decreased pulmonary function even after adjusting for confounders.
Omae et al., 1992: Four-year follow-up of effects of toluene diisocyanate exposure on the respiratory system in polyurethane foam manufacturing workers. II. Four-year changes in the effects on the respiratory system [54]	FEV_1, FVC, FEV_1/FVC	N= 57 polyurethane foam manufacturing workers (PF workers) and 24 reference workers followed for 4 years	Workers exposed to peak exposure excursions to 30 ppb or above had significantly larger average annual losses in FEV_1/FVC than expected. There were significantly larger average annual losses in some obstructive pulmonary function indices compared to the workers whose peak exposure excursion levels were lower and to the reference workers.
Pronk et al., 2007: Respiratory symptoms, sensitization, and exposure response relationships in spray painters exposed to isocyanates [16]	The prevalence of respiratory symptoms and sensitization	N= 581 workers in the spray-painting industry exposed to isocyanates	Respiratory symptoms were more prevalent in exposed workers compared to office workers. Exposure–response associations were detected for COPD-like symptoms and work-related chest tightness (prevalence ratios for an interquartile range increase in exposure of 1.3 and 2.0, respectively; $p\leq 0.05$).
Glindmeyer et al., 2004: Spray-painting and chronic airways obstruction [55]	Spirometry data: FEV_1, FVC, FEV_1/FVC	N= 240 spray-paint workers, a cross-sectional study	After adjusting for smoking and asthma symptoms, higher exposures of respirable paint aerosols and isocyanates were associated with statistically significant reduction in expiratory flowrates.

Table S7. PAHs studies on COPD.

Authors, Year and Title	Outcome Measured	Population Studied	Results
Leachi et al., 2019: Polycyclic aromatic hydrocarbons and development of respiratory and cardiovascular diseases in workers [60]	Review article, outcomes varied	Study designs and populations varied	Exposure to PAHs was associated with respiratory disorders such as decreased lung function, COPD, and respiratory symptoms.
Zhang et al., 2021: Associations and dose–response relationships between different kinds of urine	FVC, FEV_1, FEV_1/FVC	Data from the NHANES 2009-2012 database, N= 3 616	Urine PAHs may relate to changes in lung function. Lung function indices decreased with

polycyclic aromatic hydrocarbons metabolites and adult lung functions [61]			the increase of urine PAHs metabolites concentrations.
Nwaozuzu et al., 2021: Systematic review of exposure to polycyclic aromatic hydrocarbons and obstructive lung disease [62]	Review article, outcomes varied; health outcomes in adults: FEV_1, FVC	Study designs and populations varied	Positive correlations between PAHs and obstructive lung diseases and reduced respiratory function were observed.
Shiue, 2016: Urinary polyaromatic hydrocarbons are associated with adult emphysema, chronic bronchitis, asthma, and infections: US NHANES, 2011-2012 [63]	Urinary PAHs and adult self-reported respiratory health conditions	Data from the NHANES 2011-2012 database, N= 5 560	Urinary PAHs were associated with adult respiratory health conditions such as emphysema and chronic bronchitis. However, causality was not confirmed.
van der Molen et al., 2018: Association between work and chronic obstructive pulmonary disease (COPD) [7]	Review article, outcomes varied; FEV_1, FEV_1/FVC, and vapors, dusts, gases, and fumes (VDGF) exposures at work, COPD defined by GOLD-staging	Working population in different industries	Two reviews including meta-analyses were associated and showed excess risk of COPD for work-related general exposure to VDGF (summary OR 1.4, 95% CI 1.19-1.73) and to inorganic dust, with a mean difference in predicted FEV_1 of -5.7% (-8.62% to -2.71%). Exposure to VDGF at work was associated with a small but increased risk of COPD.

In the study of *van der Molen et al. (2018)* [7] there was also a study by *Fontana et al. (2017)* [9] included.

Additional note from the authors:

Some of the original articles were included in various studies, causing the results of these specific studies to be emphasised. Underneath the tables of pesticides, Cd, As, and Pb, these overlapping studies are mentioned, whereas regarding Cr, diisocyanates, and PAHs there were no overlapping original studies detected. However, regarding PAHs, the review study by Fontana et al. (2017) [9] was also included in the review by van der Molen et al. (2018) [7].

References

77. Faria, N.M.X.; Facchini, L.A.; Fassa, A.G.; Tomasi, E. Pesticides and respiratory symptoms among farmers. **2005**, *39*, 973–981, https://doi.org/10.1590/s0034-89102005000600016.
78. Hoppin, J.A.; Valcin, M.; ScD, P.K.H.; Kullman, G.J.; Umbach, D.M.; London, S.; Alavanja, M.C.; Sandler, D.P. Pesticide use and chronic bronchitis among farmers in the agricultural health study. *Am. J. Ind. Med.* **2007**, *50*, 969–979, https://doi.org/10.1002/ajim.20523.
79. Chakraborty, S.; Mukherjee, S.; Roychoudhury, S.; Siddique, S.; Lahiri, T.; Ray, M.R. Chronic exposures to cholinesterase-inhibiting pesticides adversely affect respiratory health of agricultural workers in India.. *J. Occup. Heal.* **2009**, *51*, 488–497, https://doi.org/10.1539/joh.l9070.
80. Schenker, M.B.; Stoecklin, M.; Lee, K.; Lupercio, R.; Zeballos, R.J.; Enright, P.; Hennessy, T.; Beckett, L.A. Pulmonary Function and Exercise-associated Changes with Chronic Low-Level Paraquat Exposure. *Am. J. Respir. Crit. Care Med.* **2004**, *170*, 773–779, https://doi.org/10.1164/rccm.200403-266oc.
81. de Jong, K.; Boezen, H.M.; Kromhout, H.; Vermeulen, R.; Postma, D.S.; Vonk, J.M.; The LifeLines Cohort study Pesticides and other occupational exposures are associated with airway obstruction: the LifeLines cohort study. *Occup. Environ. Med.* **2013**, *71*, 88–96, https://doi.org/10.1136/oemed-2013-101639.
82. Valcin, M.; Henneberger, P.K.; Kullman, G.J.; Umbach, D.M.; London, S.; Alavanja, M.C.R.; Sandler, D.P.; Hoppin, J.A. Chronic bronchitis among nonsmoking farm women in the agricultural health study. *J. Occup. Environ. Med.* **2007**, *49*, 574–583, https://doi.org/10.1097/jom.0b013e3180577768.
83. Rinsky, J.L.; Richardson, D.B.; Kreiss, K.; Nylander-French, L.; Freeman, L.E.B.; London, S.; Henneberger, P.K.; Hoppin, J.A. Animal production, insecticide use and self-reported symptoms and diagnoses of COPD, including chronic bronchitis, in the Agricultural Health Study. *Environ. Int.* **2019**, *127*, 764–772, https://doi.org/10.1016/j.envint.2019.02.049.
